# Medical Diseases of the War

**Published:** 1917-07

**Authors:** 


**Medical Diseases of the War.** By ARTHUR F. HURST, M.A., M.D. Oxon., F.R.C.P. Pp. 151. London: E. Arnold. 1917. Price 6s. net.

This is a most useful book, especially for those who have the care of invalid soldiers. It gives a summary of the most recent knowledge on such subjects as shell shock, dysentery, the para-typhoids and trench fever, soldier's heart, war nephritis, and gas poisoning.

The author shows, for instance, how important auto-suggestion is in the production of the various symptoms of shell shock. The attention of the patient as he recovers consciousness being fixed on one of the functions, such as speech or movement of an arm which was returning more slowly than others, he becomes convinced that it is permanently lost, with the result that a real hysterical paralysis takes place. The analysis of the various symptoms is excellently detailed, and the author reports a large number of cases of successful treatment by hypnotic suggestion. Other elements in the causation of the symptoms, such as minute hemorrhages due to monoxide poisoning from cordite, and compression of the air from high explosives, are also considered.

The sections on gas poisoning and war nephritis are too short, but the author points out the practical distinction in the latter disease from acute exacerbations of old renal disease, viz., the absence of circulatory and retinal changes, and notes the low mortality and tendency to complete recovery, in spite of the frequency of severe uræmic convulsions.

On the question of the so-called soldier's heart, the origin is referred in many cases to bacterial intoxication after slight illnesses, in others to hyperactivity of the thyroid and the other ductless glands, and in some to excessive smoking. It is important that the man should not consider himself a sufferer from heart disease, and anyone with experience in Medical Boards knows the number of cases labelled valvular disease who come up after a time of rest with no trace of murmur or other abnormality left.

The chapters on dysenteries and para-typhoids are excellent, and contain many references to recent papers. The author distinguishes the two forms of epidemic jaundice, viz. the spirochetal and the catarrhal, and denies any direct connection between the latter disease and dysentery or mild para-typhoid, to which it has been ascribed by others.

